# The effect of mutations on binding interactions between the SARS-CoV-2 receptor binding domain and neutralizing antibodies B38 and CB6

**DOI:** 10.1038/s41598-022-23482-5

**Published:** 2022-11-05

**Authors:** Jonathan E. Barnes, Peik K. Lund-Andersen, Jagdish Suresh Patel, F. Marty Ytreberg

**Affiliations:** 1 Institute for Modeling Collaboration and Innovation, University of Idaho, Moscow, ID 83843 USA; 2 Department of Biological Sciences, University of Idaho, Moscow, ID 83843 USA; 3 Department of Physics, University of Idaho, Moscow, ID 83843 USA

**Keywords:** High-throughput screening, Protein function predictions

## Abstract

SARS-CoV-2 is the pathogen responsible for COVID-19 that has claimed over six million lives as of July 2022. The severity of COVID-19 motivates a need to understand how it could evolve to escape potential treatments and to find ways to strengthen existing treatments. Here, we used the molecular modeling methods MD + FoldX and PyRosetta to study the SARS-CoV-2 spike receptor binding domain (S-RBD) bound to two neutralizing antibodies, B38 and CB6 and generated lists of antibody escape and antibody strengthening mutations. Our resulting watchlist contains potential antibody escape mutations against B38/CB6 and consists of 211/186 mutations across 35/22 S-RBD sites. Some of these mutations have been identified in previous studies as being significant in human populations (e.g., N501Y). The list of potential antibody strengthening mutations that are predicted to improve binding of B38/CB6 to S-RBD consists of 116/45 mutations across 29/13 sites. These mutations could be used to improve the therapeutic value of these antibodies.

## Introduction

SARS-CoV-2, the viral pathogen behind the COVID-19 pandemic, has battered the world population medically, economically, and socially. Infected individuals can suffer from a range of symptoms, from moderate cold-like, to severe pneumonia-like^1^. Furthermore, those infected can suffer long-term effects termed “Long-Haul COVID”, the mechanisms and extent of which is not fully understood^2^. Due to the severity and high mortality rate, vaccines were rapidly developed and deployed worldwide^3^. As the pandemic has progressed, novel variants such as Omicron^4,5^ appeared with the ability to escape protection from antibodies and potentially vaccines^6,7^.

The primary mechanism of SARS-CoV-2 cell entry is via the receptor binding domain of the Spike glycoprotein (S-RBD) that is located on the surface of the virus^8^. The S-RBD has been shown to be a successful target for antibody therapies. Dejnirattisai et al. tested monoclonal antibodies (mAbs) from patients with verified SARS-CoV-2 infections, finding that 19 of the mAbs that targeted S-RBD were potent neutralizers compared to only one that targeted the N-terminal domain^9^. Piccoli et al. explored SARS-CoV-2 immune sera, finding that 90% of the antibody neutralization was from those targeting the S-RBD^10^. For these reasons, there are a number of antibodies that have been identified and developed to target the S-RBD^11^. Of particular importance to this study are the B38 and CB6 antibodies. B38 was one of the earliest protective antibodies discovered^12^ and is currently a basis for engineering new antibodies^13^. CB6 (also known as LY-CoV016, JS016, or Etesevimab) was designed in 2020 and has been used in combination with LY-CoV555 as a treatment for patients with mild to moderate COVID-19 diesase^14,15^. These antibodies share similar epitopes suggesting that single mutations could allow escape from both B38 and CB6. When compared, B38 has five unique contacts with S-RBD (Y453, F490, G496, Q498, V503) and CB6 has three (R405, R408, G504), with the remaining 30 contacts shared between them (85% and 91% of the epitope is shared for B38 and CB6 respectively)^12,14^.

Since it has become routine practice to monitor genetic variation during outbreaks and pandemics, an aspirational goal in the fight against COVID-19 is to predict when viral evolution is outpacing the effectiveness of protective antibodies and exploring ways to retain or improve their efficacy. Antibody escape can occur for many possible reasons. For example, if a mutation significantly reduces affinity to an antibody, then it is likely to result in escape from that antibody regardless of other possible mutation effects. However, a mutation can also affect binding rates, folding efficiency, or downstream characteristics that could lead to escape without a change in affinity. Experimental work has been done to map mutations in the S-RBD that show antibody escape. Bloom and collaborators generated a complete map of mutations that escape binding with 10 monoclonal antibodies (not including B38 or CB6)^16^. Important for our study, two other studies from the Bloom lab evaluated escape mutations for CB6; one included just CB6^17^ and another considered CB6 and its cocktail with Ly-CoV555^18^. These studies used a deep mutational scanning technique to ascertain the effects of mutations in the S-RBD and binding with ACE2^19^ and led them to develop software that predict antibody escape mutations^20^.

Computationally, Teng et al. used FoldX to perform deep mutational scanning to predict stability and determine the effects on binding affinity between the S-RBD and ACE2^21^. Tsai et al. also used FoldX, MutaBind2, and mCSM-PPI2 to generate heatmaps for possible antibody escape for five antibodies bound to the S-RBD^22^. These five antibodies all bind to the S-RBD, a few of which bind to similar parts of the S-RBD as B38 and CB6. Sharma et al. used ProAffiMuSeq, mCSM, CUPSAT, and FoldX to determine relevant structural aspects for binding affinity between the S-RBD and antibodies, noting attributes like changes to Tyrosine (Y) as significant^23^. Furthermore, there have been computational studies that investigated known variants bound to B38 and CB6 and their potential escape behavior. Ray et al. used protein-graph-connectivity networks to investigate the changes in dynamics with known variants and their interaction with mAbs B38 and BD233^24^. Hendy et al. used molecular dynamics simulations to predict the binding affinity between antibodies (including B38) with the RBD considering three mutation sets matching those found in variants B1.1.7, B1.1.28, and B1.351^25^. They also explored potential mechanisms of escape. Miller et al. explored the effects of mutations specific to the recent Omicron variant using amino acid interaction networks^26^. They tested binding with a few antibodies including CB6. Laurinini et al. performed alanine scanning to investigate potential escape mutations for antibodies including CB6 finding 90% agreement to experimental results^27^. While such studies are informative at identifying sites that are significant for binding, they do not provide information specific to individual mutations. We seek to build on these previous studies to provide a comprehensive list of escape mutations for B38 and CB6 by predicting binding affinity changes due to all possible amino acid changes.

In this work, we use an approach previously developed in our group to generate watchlists of mutations for ebolavirus^28,29^ using a fast empirical method, FoldX^30^, combined with rigorous molecular dynamics (MD) simulations (termed “MD + FoldX”). We also generated results using PyRosetta software. We applied these methodologies to the S-RBD in a complex with two antibodies, B38 and CB6 to predict a large number of possible mutations of S-RBD that can disrupt antibody binding and allow escape. The appearance of mutations in this watchlist in an emerging variant will be evidence that treatments using these antibodies may be less effective. Additionally, we identified mutations in the antibodies that are predicted to improve their binding to the S-RBD. These mutations may strengthen the therapeutic effect of the antibody against COVID-19.

## Methods

We employed the MD + FoldX approach developed in our group to build a watchlist of antibody escape mutations^28,29,31^. Following this approach, we first carried out short MD simulations using the 3D structures of the antibody-S-RBD complexes (for both B38 and CB6) as inputs to sample conformational flexibility. The snapshots from the MD simulations were then used as inputs for FoldX to perform mutational scanning and generate averaged predictions for relative binding (ΔΔG_bind_) and folding (ΔΔG_fold_) free energy changes due to all possible mutations at sites near the binding interface. To overcome the limitation of FoldX software and to improve the overall escape mutation prediction accuracy, we also used the PyRosetta program. We used the built-in conformational sampling procedure in PyRosetta, and its knowledge-based scoring function to estimate ΔΔG_bind_ and ΔΔG_fold_ values. Both MD + FoldX and PyRosetta approaches were also used to scan all possible mutations of B38 and CB6 to identify mutations that may improve their binding to the S-RBD. Mutation sites were chosen to be those within 10 Å of the binding interface based on the energy minimized structures. This is reasonable since both FoldX and PyRosetta are expected to only capture local effects. The mutation sites for S-RBD, for example, were determined by selecting α-carbons on the S-RBD that were within 10 Å of any other atom belonging to either the heavy or light antibody chains.

### System determination and preparation

**Figure 1:**
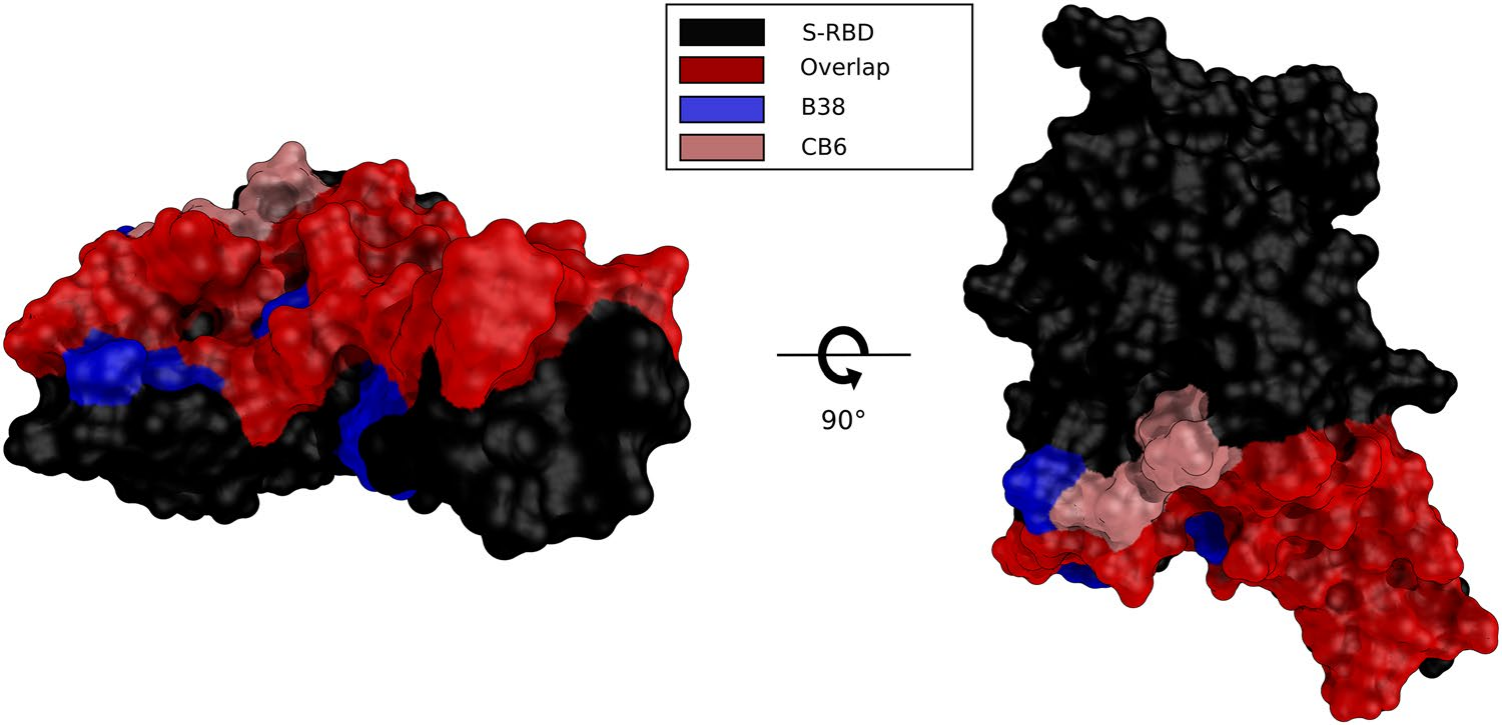
Comparison of epitopes for antibodies B38 and CB6 on S-RBD. Red (overlapping epitope), Blue (B38) and Salmon (CB6) colors are used to compare epitope regions on the 3-D structure of the S-RBD (black) shown in surface representation. Two viewpoints of the S-RBD structure are shown to provide full context.

To generate a watchlist of potential antibody escape mutations for the S-RBD and identify mutations that could improve antibody binding affinity, we selected two S-RBD-antibody complexes: B38 and CB6. These complexes have Protein Data Bank^32^ identifiers of 7BZ5 and 7C01 corresponding to antibodies B38 and CB6, respectively. Figure 1 shows the epitopes of B38 and CB6 bound to the S-RBD, highlighting the overlapping regions.

Structures were prepared and solvated using the OpenMM-setup package. The AMBER14 forcefield was used to generate protein topology files along with the default TIP3P-FB water model. Only the protein chains were retained with all heterogens and water removed. For the B38 complex, the missing HIS at site 519 in chain A was added since it is an internal loop; other missing residues were not added since they are part of the termini and located away from the binding interface. The CB6 complex has missing residues located at the termini only, so none were modeled.. Heavy atoms were added for the B38 complex (LYS528, SER217, CYS215) and the CB6 complex (PRO527, GLU218, GLU215). The following protocol was then applied for all simulations. Hydrogens were added corresponding to a pH of 7.0. A rhombic dodecahedral water box was used with a padding distance of 1.2 nm. Sodium and chloride atoms were added to neutralize the net charge of the systems with an ionic strength of 0.15 M. MD simulations were then carried out using OpenMM^33^ with the following protocol. The systems were first minimized for 2000 steps using *minimizeEnergy*. Next, we performed NVT annealing with position restraints for a total of 1 ns starting at 10 K and stopping at 310 K with a ΔT of 3 K and 5000 steps at each temperature. NPT equilibration was then performed for 1 ns at 310 K, with a pressure of 1 bar maintained via the Monte Carlo barostat with an interval of 25 steps. For all simulations the Langevin integrator was used and the time step set to two fs. The only constraints were hydrogen bonds. Production simulations were then run for 100 ns with the same parameters except for the barostat interval adjusted to every 100 steps, with snapshots taken every 1 ns for a total of 100 snapshots to be used for analysis. For binding affinity calculations, we then performed a total of two simulations (S-RBD-B38 and S-RBD-CB6 complexes). For folding stability calculations, we performed five simulations (S-RBD, B38 H, B38 L, CB6 H, and CB6 L) where H and L denote the heavy and light chains for each antibody, respectively.

### Mutation scanning

Following the protocols used for the Ebola watchlist and watchlist expansion studies^28,29,31^, FoldX was used to estimate the folding stability of S-RBD and the binding affinity between S-RBD and the two antibodies using 100 MD snapshots from each simulation as inputs. Each snapshot was first prepared by running the *RepairPDB* command six times in succession to ensure convergence of the potential energy. The *BuildModel* command was then used to generate mutations to all possible 19 amino acids at all sites within 10 Å of the binding interface. For completeness, we considered the union of sites between the S-RBD-B38 complex and the S-RBD-CB6 complex consisting of 75 sites for the S-RBD, 51 sites for antibody B38, and 55 sites for antibody CB6. Mutations were introduced on the S-RBD for predicting antibody escape mutations (1425 total mutations) and on both the antibodies to identify antibody strengthening mutations (1349 mutations for B38, 1407 for CB6). *AnalyseComplex* was then used to predict ΔG_bind_ values for the wildtype and mutants and the difference was calculated to obtain ΔΔG_bind_ values. This process was repeated for the 100 snapshots, and then averaged to generate the final predicted ΔΔG_bind_. Similarly, to predict ΔΔG_fold_ values, the MD snapshots for each individual protein chain were analyzed using the *Stability* command and averaged. In addition to MD + FoldX, PyRosetta-4^34^ was used to estimate the differences in binding and folding stability scores between mutant and wildtype structures. The ref2015 forcefield^35^, a knowledge-based energy function, was used for all PyRosetta calculations and procedures. The *flexddG* protocol^36^ implemented in PyRosetta was used to estimate ΔΔG_bind_ and ΔΔG_fold_ values and is briefly described here. The experimental structures were used as inputs and they were first relaxed with PyRosetta’s *FastRelax* protocol^37^. Here, we used the experimental structures (not the MD snapshots) since protocols for PyRosetta already generate conformational ensembles, in contrast to FoldX protocols that use a single structure and hence benefits from additional conformational sampling. A conformational ensemble of 50 structures was then generated using PyRosetta’s *backrub* protocol^38^ over 50 iterations, to sample backbone torsions within 8 Å of the mutation site. For each structure, all possible mutations for a given residue were then introduced, followed by global repacking of side chains using the 2010 Dunbrack rotamer library^39^. The structures then underwent energy minimization using the *lbfgs_armijo_nonmonotone* algorithm with a tolerance of 1e-5 and a convergence threshold of 0.5 kcal/mol. The wildtype structures were also refined with the sidechain packing and energy minimization using the same parameters. To calculate binding ΔG values, we computed the total energy of the bound complex and subtracted the energies of the separated antibody and antigen structures then averaged the results. For both binding and folding stability, the differences between the mutated and wildtype structure energies were taken to obtain ΔΔG_bind_ and ΔΔG_fold_, respectively. These ΔΔG_bind_ and ΔΔG_fold_values were then averaged across the 50 conformations to get the final values. To our knowledge PyRosetta has not been as thoroughly tested as MD + FoldX for antibody-antigen systems, hence we ran a test using the antibody-antigen benchmarking systems from Gonzales et al. to compare its accuracy for this purpose. We found that for two of the test systems, PyRosetta performed very similarly to FoldX (within 2% or better). One of the test systems performed significantly better, and we found poor performance for the other half of the systems, primarily driven by a handful of outliers. The full details are shown in Supplementary Figure S1.

## Results and discussion

The goal of this study is to develop both a watchlist of potential antibody escape mutations against two antibodies that interact with the S-RBD of SARS-CoV-2, and a list of possible antibody strengthening mutations. Watchlist mutations are amino acid changes in S-RBD that either MD + FoldX or PyRosetta predict to disrupt its binding to the B38 or CB6 antibodies, hence potentially reducing therapeutic efficacy of these antibodies. Similarly, antibody strengthening mutations are mutations in the antibodies that are predicted to strengthen their binding interaction to S-RBD, hence potentially increasing the therapeutic efficacy of the antibodies.

For a mutation to be part of our watchlist, i.e., be a potential escape mutation, we used the cutoff of ΔΔG_bind_ > 2.0 kcal/mol from our previous studies to classify binding disruption^28,29,31^. For antibody strengthening, our cutoff of ΔΔG_bind_ < − 0.5 kcal/mol was chosen to be consistent with the standard deviation determined during the validation study of FoldX that is reported as 0.46 kcal/mol^30^. For folding, we used a cutoff of -3.0 kcal/mol < ΔΔG_fold_ < 3.0 kcal/mol to define when a protein can still fold. All cutoffs were purposely chosen to be inclusive rather than conservative given the uncertainty of the calculations. Finally, note that the *REF15* energy function we used in PyRosetta provides results expressed in kcal/mol not the previously-used Rosetta Energy Units^35^. This allows us to use the same cutoffs for both FoldX and PyRosetta predictions. We note that FoldX and PyRosetta use different strategies to estimate affinities and so we do not expect full agreement. FoldX uses a semi-empirical forcefield and relaxes the input structures using a proprietary algorithm termed *repairPDB*. PyRosetta performs Monte Carlo minimization of the input structure, generates a conformational ensemble and then uses a physics-based scoring function for binding affinity predictions of the ensemble. Our union dataset includes results from both methods to minimize the risk of leaving out possible escape mutations.

**Figure 2:**
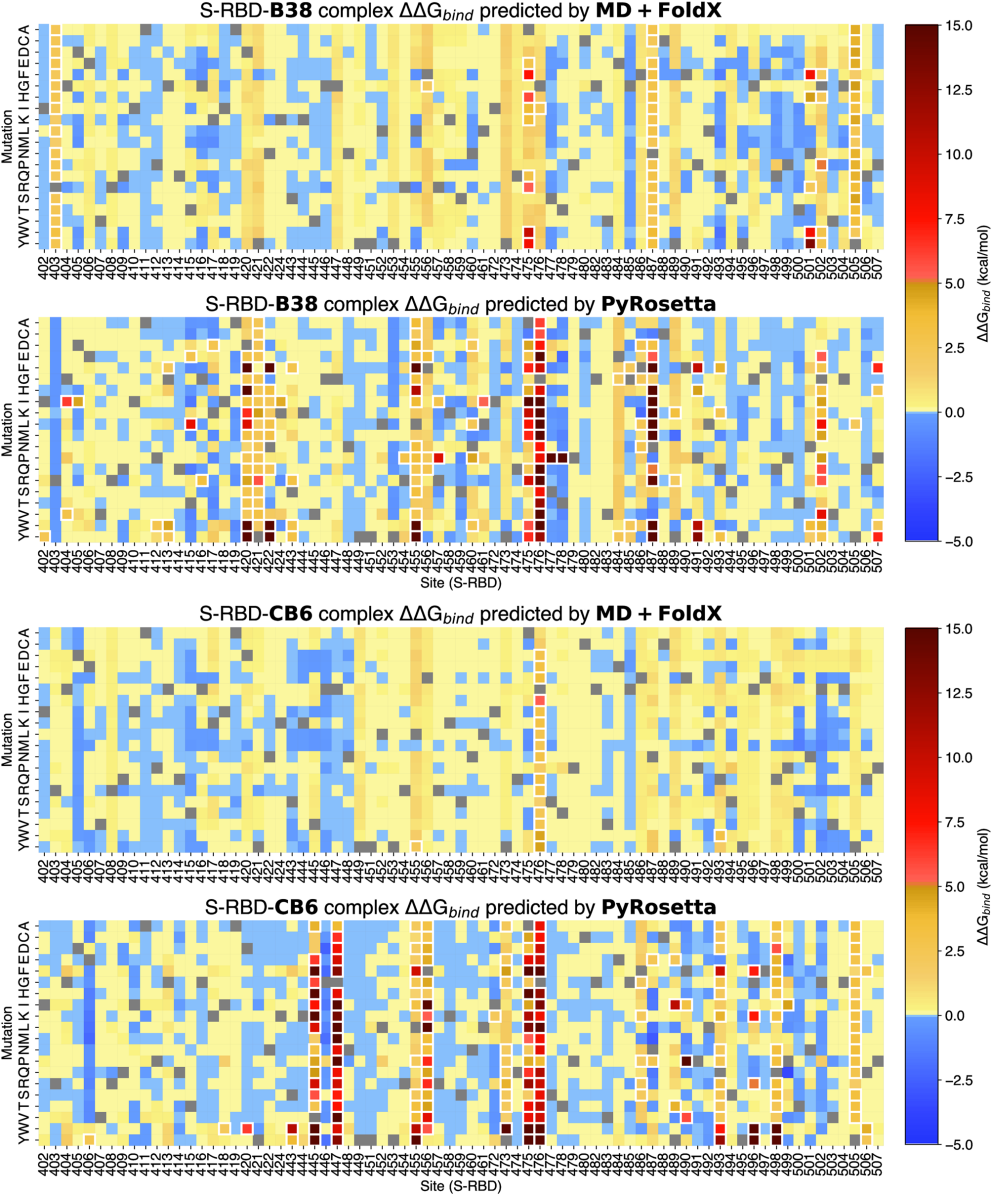
Heatmaps for S-RBD mutations showing PyRosetta and MD + FoldX predicted ΔΔG_bind_ values for all possible 19 mutations at each site on the epitopes of B38 (top) and CB6 (bottom) antibodies. Blue indicates mutations that lead to stronger binding (ΔΔG_bind_ < 0), yellow and red indicates binding disruption (ΔΔG_bind_ > 0). The white highlighted data points are cases where the ΔΔG_bind_ values are above the cutoff, hence are predicted to be antibody escape mutations (ΔΔG_bind_ > 2 kcal/mol). This figure was generated using the Python programming language v3.10.8 (Python software foundation, https://python.org) and the matplotlib plotting module v3.5.2^40^.

The heat maps in Fig. 2 show S-RBD-antibody binding affinity predictions from MD + FoldX and PyRosetta due to mutations in S-RBD (full results are available in Supplemental Dataset S1). Each method predicts possible escape mutations at specific sites. For B38, MD + FoldX predicts a total of 73 potential escape mutations at eight sites in S-RBD. More than 50% of these belong to sites R403, A475, N487, N501, and Y505. For CB6, MD + FoldX predicts 16 escape mutations at three sites, with site G476 containing the most. PyRosetta predicts a much larger escape mutation dataset with 165 and 187 escape mutations for B38 and CB6, respectively. We note that for some sites in B38 (e.g., D420 and Y421), MD + FoldX predicts destabilizing mutations but none that exceed our cutoff (ΔΔG_bind_ > 2.0 kcal/mol) to qualify as antibody escape mutations. By contrast, PyRosetta predicts 16 antibody escape mutations for sites D420 and Y421. Similarly, this can also be seen in the case of CB6 for sites L455 and F456.

We believe that the most valuable results in this study are the union of the MD + FoldX and PyRosetta datasets; the union dataset limits possible false exclusio1n of mutations on a watch list. To motivate our reasoning behind using the union dataset, consider that Fig. 2 shows that MD + FoldX predicts N501Y as a possible escape mutation for B38, however PyRosetta does not predict it for either antibody. N501Y is a known mutation present in a number of variants of concern, both on its own and in combination with others^41–44^. Additionally, it has been shown to be key in epistatic interactions associated with increased infectivity^45^. We note that PyRosetta still predicts a mutation at site 501, but only N501W for B38.

**Figure 3:**
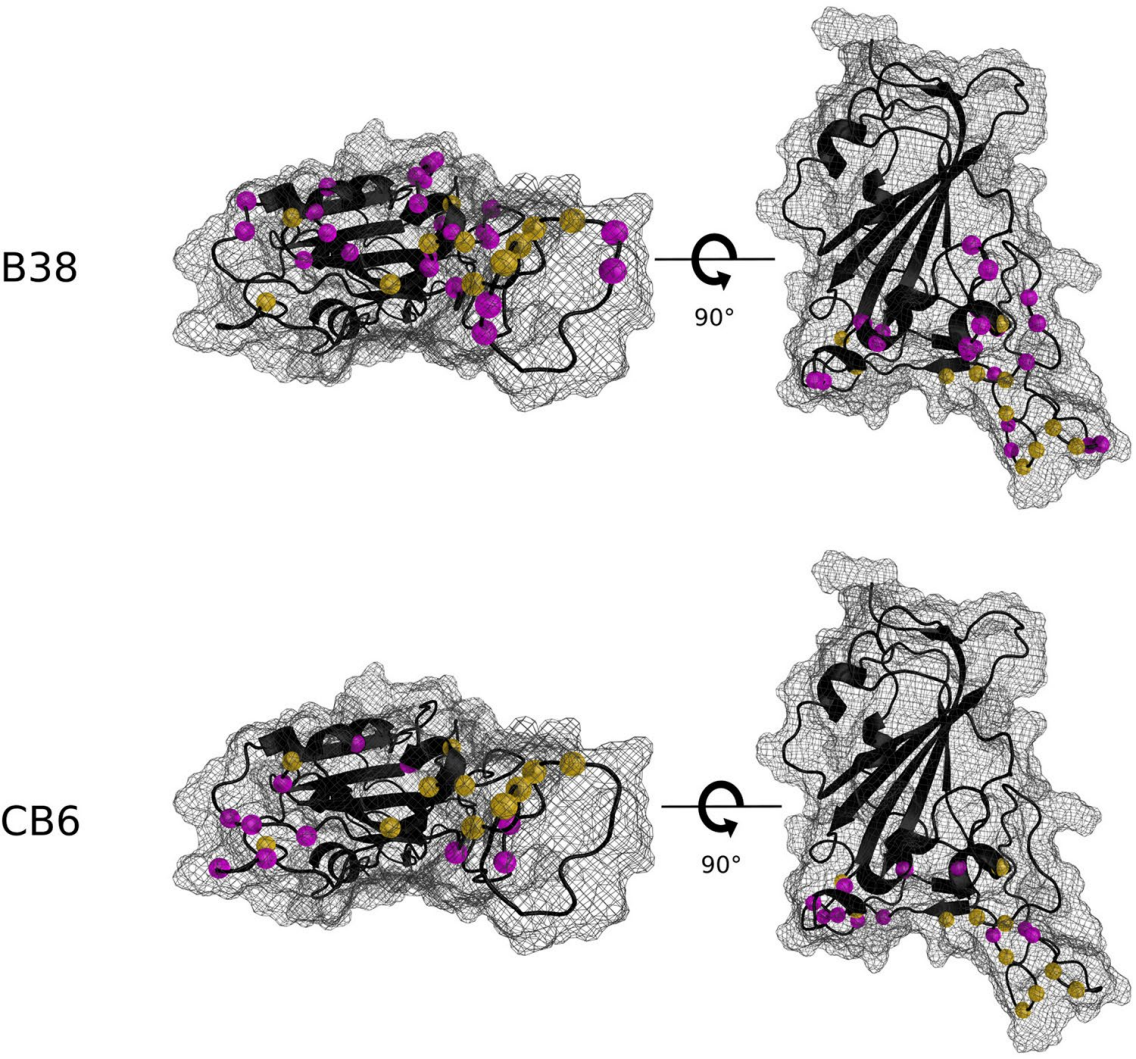
The S-RBD amino acid sites predicted to contain antibody escape mutations (magenta) by MD + FoldX or PyRosetta (i.e., all sites listed in Table 1). The S-RBD sites that are predicted to harbor antibody escape mutations for both B38 and CB6 are shown in gold. For both B38 (top) and CB6 (bottom) two views are shown to provide context. The S-RBD is indicated in black, with the surface shown as a mesh.

The union dataset (i.e., includes both MD + FoldX and PyRosetta) is shown in Table 1 and Fig. 3. This consists of 211 predicted escape mutations (ΔΔG_bind_ > 2.0 kcal/mol) across 35 sites for B38 and 186 mutations across 22 sites for CB6. There are 178 mutations within this combined watchlist that are predicted to not fold properly, however, we still include them in an effort to be as inclusive as possible. We also note that if a mutation does indeed significantly disrupt the protein fold, then it will not appear in a natural population. The sites with the highest concentration of potential escape mutations for B38 are R403, D420, Y421, L455, A475, G476, N487, G502, and Y505 (contain 51% of the predicted escape mutations). For CB6 these are V445, G447, L455, F456, Y473, A475, G476, Q493, Q498, and Y505 (contain 85% of the predicted escape mutations). We predict 11 sites to harbor antibody escape mutations for both antibodies, all within the shared epitope region. Table 2 shows the frequency of the mutated amino acids predicted to allow antibody escape. We see that the most common mutations for both B38 and CB6 are to tryptophan (W) – a large side chain – intuitively likely to disrupt function, especially if the side chain is small in the wildtype protein. Furthermore, we see that smaller side chains like alanine and glycine appear less frequently in the table.

**Table 1:**
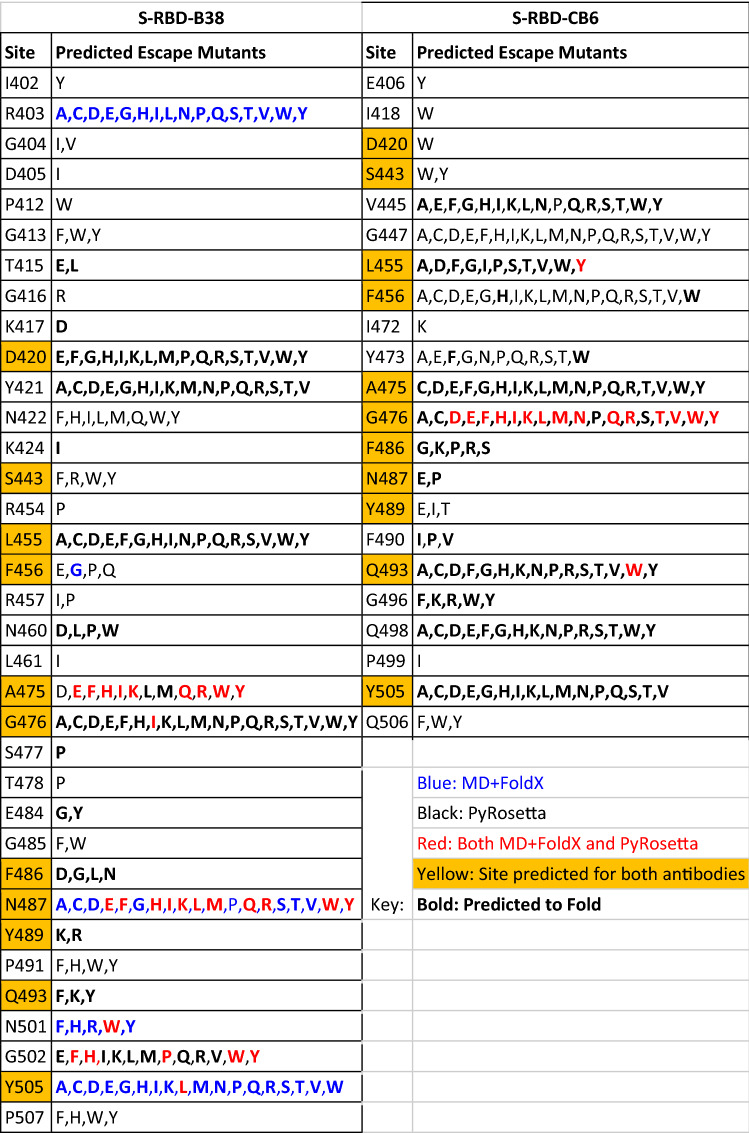
Union dataset of predicted escape mutations for B38 and CB6 antibodies based on both MD + FoldX and PyRosetta simulations

The left and right tables show results for B38 and CB6, respectively. For each subtable, the left column is the S-RBD site and the right contains the mutations predicted by either MD + FoldX and/or PyRosetta to lead to antibody escape. Predicted escape mutations are colored based on whether both methods predict escape (red) , only MD + FoldX predicts escape (blue), or only PyRosetta predicts escape (black). The yellow highlighted sites are those predicted to contain antibody escape mutations for both antibodies. Bold font denotes mutations that are predicted to allow folding

There are currently no large-scale experimental studies evaluating antibody escape for B38. For CB6, however, there are experimental results that we can use to compare with our predictions. Starr et al. used deep mutational scanning to build a map of mutations in S-RBD and measured how these mutations modify the ability of S-RBD to bind CB6^17^ They measured the escape fraction for nearly all possible mutants in the S-RBD. Mutations that were excluded were filtered out for having low sequencing counts, for low binding with ACE2 (based on the binding score of RaTG13 to human ACE2, lowest known affinity capable of cell entry), or for being a variant with poor RBD expression. Of the 186 mutations in our watchlist, 48 are excluded from the Starr et al. study (see Supplemental Table S1). Of those excluded, we predict 39 to fold (−3.0 kcal/mol < ΔΔG_fold_ < 3.0 kcal/mol). The binding results from Starr et al. were provided in escape fraction, a metric that ranges from 0 (no observed escape, antibody always bound) to 1 (a decrease in binding frequency by 100 × or more). While we cannot directly compare escape fraction to ΔΔG_bind_, we can safely assume that a mutation with a large (actual, not predicted) ΔΔG_bind_ has a higher chance to escape and hence a larger escape fraction. Note that the converse is not true, that is, high escape fraction does not imply large ΔΔG_bind_ since mutations can escape antibody binding for a variety of reasons other than reduced affinity (e.g., modified binding rates, misfolding, etc.). Comparing our union CB6 dataset with Starr et al. we find that 20% (38) of our predicted escape mutations correspond to an escape fraction of 0.5 or greater with the majority falling below 0.1. We next looked more closely at the subset of mutations that are in agreement between our study and Starr et al., that is mutations we predict to escape that they determined to have an escape fraction greater than 0.5. For PyRosetta, we found that all 38 mutations have ΔΔG_bind_ values above the cutoff. In addition, the mutation with the largest predicted ΔΔG_bind_ of 44.52 kcal/mol corresponded to an escape fraction of 0.98. For FoldX, only four of the 38 mutations are above the cutoff, and the largest ΔΔG_bind_ is 3.23 kcal/mol corresponding to an escape fraction of 0.871. We note that there are two additional mutations in this subset, both at site L455, where FoldX predicted ΔΔG_bind_ values just below our cutoff (1.34 and 1.59 kcal/mol) that would have been excluded from our watchlist if we had only considered FoldX results. Finally, if we compare our results with Starr et al. at the site level, we see about 50% agreement (Supplemental Table S1). In some of these sites 50% of mutations are predicted to escape: L455, F456, Y473, A475, and G476. To summarize this comparison, we believe that the discrepancies between our results and the Starr et al. data highlight the need for more accurate modeling approaches and techniques.

We have also identified sites and mutations relevant to recent variants of concern. Predominant variants of concern include the UK variant (B.1.1.7; N501Y), South African variant (B1.351; N501Y, E484K, K417N, L18F, A701V), Brazilians (P1/P2; N501Y, E484K, K417T, D614G, V1176F), Californians (B.1.429/7; L452R, W152C, S13I), and Indian (Delta) (B.1.617.1, B.1.617.2/B.1.617.3; E484Q, L452R, P681R)^46^. N501Y is common in these variants of concern and is consistent with our findings, is a known antibody escape mutation, and has been shown to reduce efficacy of B38^47^. Our methods indicate E484 is also a site of significance; present in the South African, Brazilian, and Delta variants. The most recent variant, Omicron, has mysterious origins as it is a departure from all previously determined lineages^48^. Omicron consists of a large number of mutations (62 vs. 45 in other variants on average)^49^. It has also been noted to escape vaccines and other antibody treatments^6,50^. It consists of a large number of mutations in the S-RBD: G339D, S371L, S373P, S375F, K417N, N440K, G446S, S477N, T478K, E484A, Q493R, G496S, Q498R, N501Y, Y505H^51^. Of these, we identified N501Y and Y505H in B38 and Q493R, Q498R, and Y505H as CB6 escape mutations. While they were not the known mutations of significance, we did identify K417D, S477P, and T478P. We cannot expect total overlap with our findings since the Omicron variant contains a large number of mutations likely working in concert, and with likely epistatic effects^45^.

**Figure 4:**
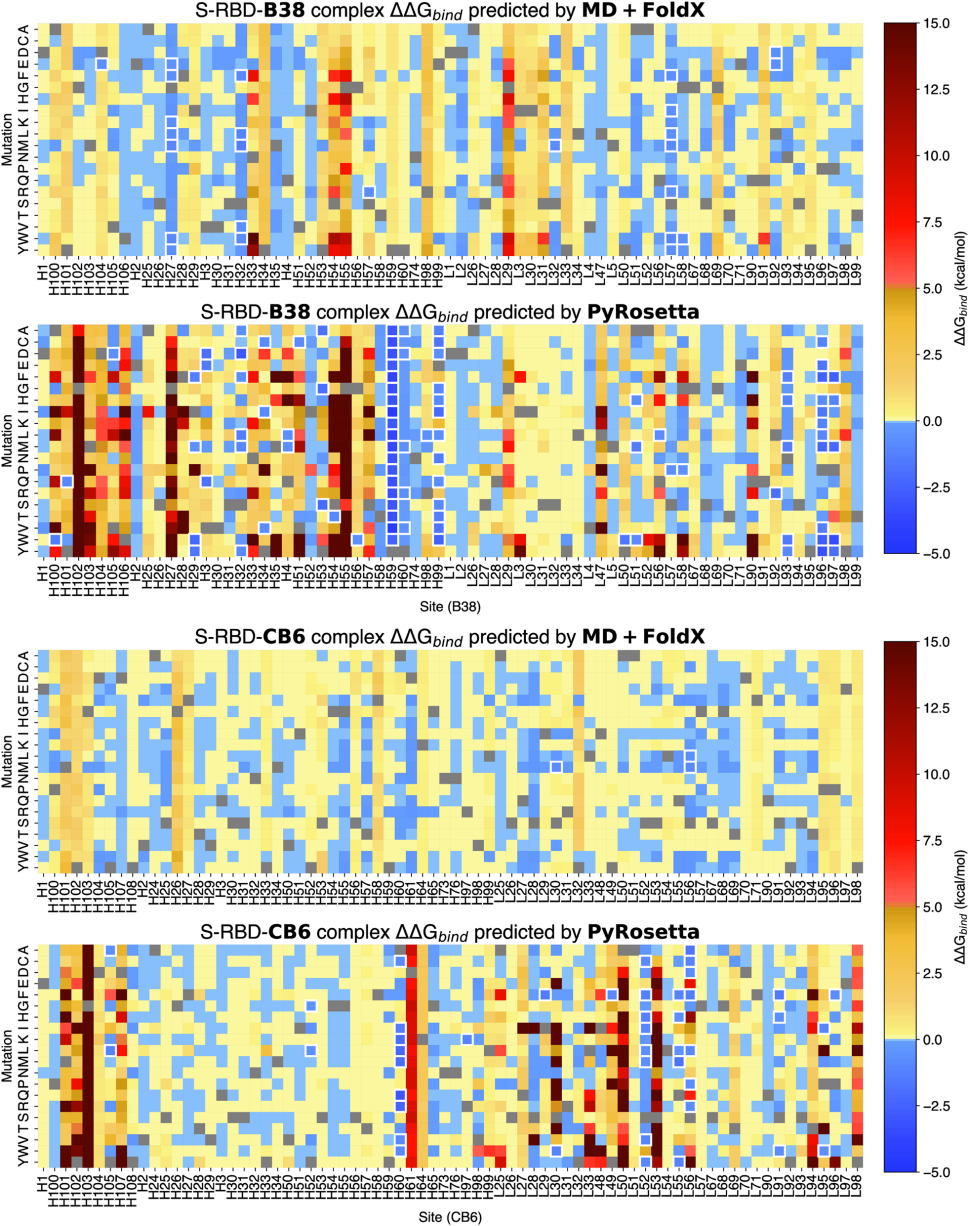
Heatmaps showing how mutations of B38 (top) and CB6 (bottom) modify binding to S-RBD. For each antibody, both MD + FoldX predictions (top) and PyRosetta predictions (bottom) are shown. Blue indicates stronger binding (ΔΔG_bind_ < 0), yellow and red indicates binding disruption (ΔΔG_bind_ > 0). Mutations predicted to be antibody strengthening (ΔΔG_bind_ <  − 0.5 kcal/mol) are highlighted in white. This figure was generated using the Python programming language v3.10.8 (Python software foundation, https://python.org) and the matplotlib plotting module v3.5.2^40^.

Using the same MD + FoldX and PyRosetta protocols, we also built a list of B38 and CB6 antibody mutations that could strengthen their interaction with the S-RBD (Fig. 4, Table 3). The figure shows a heatmap of antibody mutations and their predicted effect on binding S-RBD (full results are available in Supplemental Dataset S2). The table shows predicted antibody strengthening mutations based on our cutoff (ΔΔG_bind_ < − 0.5 kcal/mol) with those predicted to allow folding in bold (− 3.0 kcal/mol < ΔΔG_fold_ < 3.0 kcal/mol). We found 116 mutations across 29 sites and 45 mutations across 13 sites for B38 and CB6 respectively. There are currently no experimental studies detailing mutations in these antibodies and their effects on binding to the S-RBD, but B38 is currently being used to engineer improved antibodies^13^. We also note that computational studies such as ours have been used in the past to design and improve on existing antibodies for other antigens by performing single mutation mutagenesis and evaluating the effects on binding affinity^52^. We hope these results can serve as a reference for further analysis and future antibody design.

**Table 2:** Residue type frequencies for predicted escape mutations

B38		CB6	
Mutation	Frequency	Mutation	Frequency
W	17	W	14
Y	16	P	13
F	15	K	11
I	15	T	11
P	14	Y	11
H	13	E	10
R	12	F	10
E	11	I	10
L	11	R	10
Q	11	S	10
D	10	A	9
G	9	G	9
K	9	N	9
V	9	D	8
M	8	H	8
S	7	V	8
A	6	C	7
C	6	Q	7
N	6	L	6
T	6	M	5

B38 and CB6 are shown left and right respectively. The mutated residue is indicated in the left subcolumn for each and the frequency it occurs in our escape dataset is shown on the right.

**Table 3:**
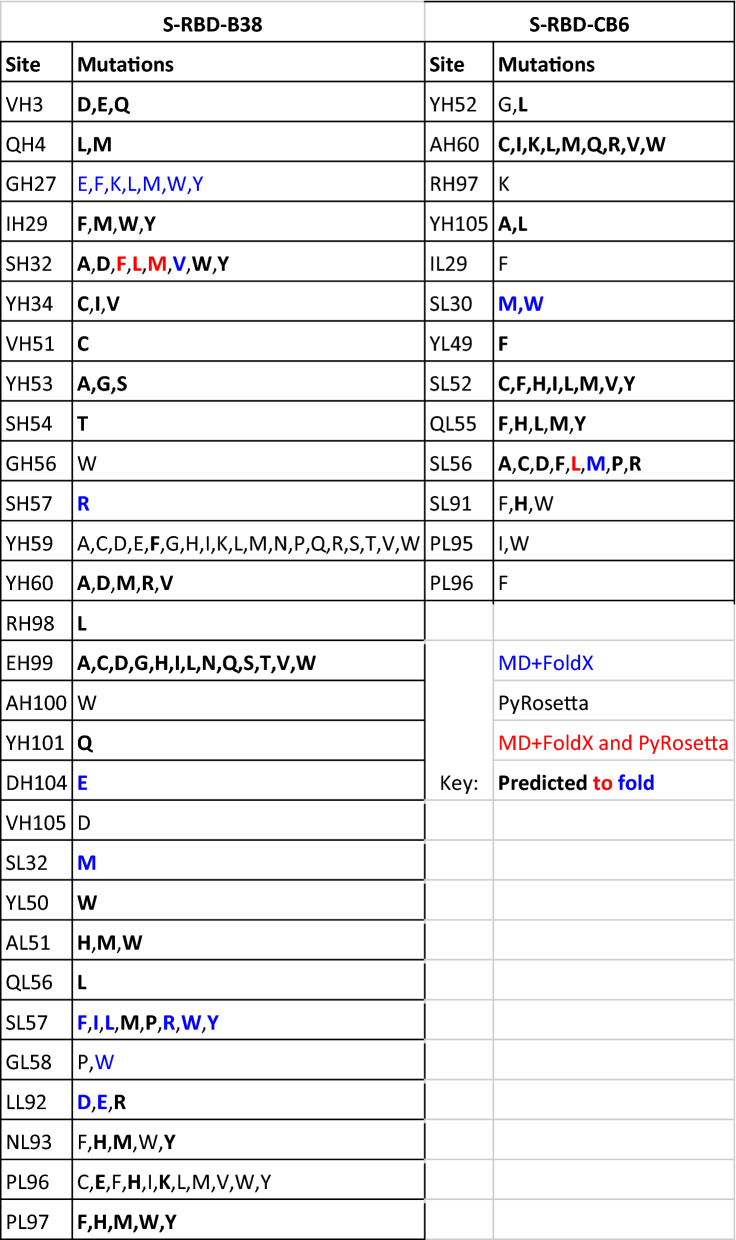
Union of the individual datasets for predicting antibody strengthening mutations from MD + FoldX and PyRosetta.

The left table contains those predicted for B38, and right is for CB6. The left column in each subtable is the site and the right column shows the list of mutations predicted to strengthen the binding interaction between the S-RBD and the antibody. Predicted strengthening mutations are colored based on whether both methods predict strengthening (red), only MD + FoldX predicts strengthening (blue), or only PyRosetta predicts strengthening (black). Bold font denotes mutations that are predicted to allow folding.

Our choice of using MD + FoldX was motivated by our previous studies^28,29^. MD + FoldX was compared to eight other methods and shown to have the best accuracy for antibody-antigen complexes^31^. Another recent study experimentally validated this approach, demonstrating that six of eight escape mutations predicted by MD + FoldX showed decreased neutralization in respiratory syncytial virus^53^. To provide a point of comparison and include mutations that might be “missed” by MD + FoldX, we also used PyRosetta. A limitation of our study, and indeed all studies that consider only single point mutations, is the possibility of epistasis. Computational methods that may be capable of predicting epistatic effects are rigorous and computationally expensive. Fast methods such as FoldX and PyRosetta are trained or referenced by datasets consisting of single point mutations, hence tend to predict additivity in the case of two or more mutations. It is already understood that epistasis is present in SARS-CoV-2^54^. Furthermore, emerging variants of concern are frequently due to multiple mutations, such as Omicron with 62 mutations throughout the genome ^49^. While well beyond the scope of the current report, it would be beneficial to future studies to develop rapid methods that are capable of accurately predicting the effects due to multiple mutations.

Our choices of cutoffs have a significant effect on results. If we consider sites and mutations from experimental data that have a strong antibody escape effect (like E484K in the Chakraborty study)^47^ we find the mutation does have a positive ΔΔG_bind_ for both FoldX (0.22 kcal/mol, well below cutoff and within FoldX error) and PyRosetta (1.81 kcal/mol). If our cutoff were 1.5 kcal/mol instead this mutation would have been included. However, this cutoff choice would also include a large set of other mutations that may or may not exhibit escape. While any choice of cutoff is inherently arbitrary, we have attempted to choose reasonable values based on previous studies and the inherent error in the calculations. For example, if we chose an antibody strengthening cutoff of ΔΔG_bind_ < 0 kcal/mol this would lead to 757 mutations across 69 sites for B38 and 773 mutations across 70 sites for CB6, of which 85% + of the results would be within the error for FoldX. If we consider known escape variants such as K417N for CB6, our results predict a positive ΔΔG_bind_ (0.566 kcal/mol and 0.0046 kcal/mol for FoldX and PyRosetta, respectively) meaning our results predict possible escape, but the mutations were not included in our watchlist since these values are below our threshold.

## Conclusions

In this study, we investigated how single amino acid mutations affect binding between the S-RBD of SARS-CoV-2 and two antibodies with similar epitopes, B38 and CB6. We considered both how mutations in S-RBD could lead to antibody escape (termed “watchlist mutations”), and how mutations in the antibodies could improve binding to the S-RBD (termed “antibody strengthening mutations”). Our watchlist for potential antibody escape (Table 1) from B38 consists of 211 mutations across 35 sites, and from CB6 consists of 186 mutations across 22 sites. Some of these mutations have been previously identified as significant in observed populations (e.g., N501Y). Our list of potential antibody strengthening mutations (Table 3) for B38 consists of 116 mutations across 29 sites, and for CB6 consists of 45 mutations across 13 sites. Our watchlist provides predictions for possible reductions in efficacy of B38 and CB6 antibodies in treating some strains of SARS-CoV-2. Similar methods could be used to predict the efficacy of other therapeutics or for other variants. Furthermore, our antibody strengthening mutation list could be used to potentially improve existing therapeutic antibodies. Finally, our comparison to the Starr et al. study highlights the need for more accurate computational methods for predicting binding affinity changes due to mutations.

## Supplementary Information

Supplementary Information 1.

Supplementary Information 2.

Supplementary Information 3.

## Data Availability

Datasets generated for this work are publicly available and provided in full as supplemental datasets S1 and S2.

## References

[1] Baj J (2020). COVID-19: Specific and non-specific clinical manifestations and symptoms: The current state of knowledge. J. Clin. Med..

[2] Mehandru S, Merad M (2022). Pathological sequelae of long-haul COVID. Nat. Immunol..

[3] Creech CB, Walker SC, Samuels RJ (2021). SARS-CoV-2 vaccines. JAMA.

[4] Khandia R (2022). Emergence of SARS-CoV-2 omicron (B.1.1.529) variant, salient features, high global health concerns and strategies to counter it amid ongoing COVID-19 pandemic. Environ. Res..

[5] He X, Hong W, Pan X, Lu G, Wei X (2021). SARS-CoV-2 omicron variant: Characteristics and prevention. MedComm.

[6] Planas D (2022). Considerable escape of SARS-CoV-2 omicron to antibody neutralization. Nature.

[7] Cao Y (2022). Omicron escapes the majority of existing SARS-CoV-2 neutralizing antibodies. Nature.

[8] V’kovski P, Kratzel A, Steiner S, Stalder H, Thiel V (2021). Coronavirus biology and replication: implications for SARS-CoV-2. Nat. Rev. Microbiol..

[9] Dejnirattisai W (2021). The antigenic anatomy of SARS-CoV-2 receptor binding domain. Cell.

[10] Piccoli L (2020). Mapping neutralizing and immunodominant sites on the SARS-CoV-2 spike receptor-binding domain by structure-guided high-resolution serology. Cell.

[11] Li D, Sempowski GD, Saunders KO, Acharya P, Haynes BF (2022). SARS-CoV-2 neutralizing antibodies for COVID-19 prevention and treatment. Annu. Rev. Med..

[12] Wu Y (2020). A noncompeting pair of human neutralizing antibodies block COVID-19 virus binding to its receptor ACE2. Science.

[13] Li Z (2022). An engineered bispecific human monoclonal antibody against SARS-CoV-2. Nat. Immunol..

[14] Shi R (2020). A human neutralizing antibody targets the receptor-binding site of SARS-CoV-2. Nature.

[15] Gottlieb RL (2021). Effect of bamlanivimab as monotherapy or in combination with etesevimab on viral load in patients with mild to moderate COVID-19: A randomized clinical trial. JAMA.

[16] Greaney AJ (2021). Complete mapping of mutations to the SARS-CoV-2 spike receptor-binding domain that escape antibody recognition. Cell Host Microbe.

[17] Starr TN (2021). Prospective mapping of viral mutations that escape antibodies used to treat COVID-19. Science.

[18] Starr TN, Greaney AJ, Dingens AS, Bloom JD (2021). Complete map of SARS-CoV-2 RBD mutations that escape the monoclonal antibody LY-CoV555 and its cocktail with LY-CoV016. Cell Rep. Med..

[19] Starr TN (2020). Deep mutational scanning of SARS-CoV-2 receptor binding domain reveals constraints on folding and ACE2 binding. Cell.

[20] Greaney AJ, Starr TN, Bloom JD (2022). An antibody-escape estimator for mutations to the SARS-CoV-2 receptor-binding domain. Virus Evolut.

[21] Teng S, Sobitan A, Rhoades R, Liu D, Tang Q (2021). Systemic effects of missense mutations on SARS-CoV-2 spike glycoprotein stability and receptor-binding affinity. Brief. Bioinform..

[22] Tsai, K.-C., Lee, Y.-C. & Tseng, T.-S. Comprehensive deep mutational scanning reveals the immune-escaping hotspots of SARS-CoV-2 receptor-binding domain targeting neutralizing antibodies. *Front. Microbiol.* **12**, (2021). 10.3389/fmicb.2021.698365 PMC8319916 34335530

[23] Sharma D, Rawat P, Janakiraman V, Gromiha MM (2022). Elucidating important structural features for the binding affinity of spike - SARS-CoV-2 neutralizing antibody complexes. Prot. Struct. Funct. Bioinform..

[24] Ray D, Quijano RN, Andricioaei I (2022). Point mutations in SARS-CoV-2 variants induce long-range dynamical perturbations in neutralizing antibodies. Chem. Sci..

[25] Hendy M, Kaufman S, Ponga M (2021). Molecular strategies for antibody binding and escape of SARS-CoV-2 and its mutations. Sci. Rep..

[26] Miller NL, Clark T, Raman R, Sasisekharan R (2022). Insights on the mutational landscape of the SARS-CoV-2 omicron variant receptor-binding domain. Cell Rep. Med..

[27] Laurini E, Marson D, Aulic S, Fermeglia A, Pricl S (2021). Molecular rationale for SARS-CoV-2 spike circulating mutations able to escape bamlanivimab and etesevimab monoclonal antibodies. Sci. Rep..

[28] Miller CR (2016). Initiating a watch list for Ebola virus antibody escape mutations. PeerJ.

[29] Patel JS, Quates CJ, Johnson EL, Ytreberg FM (2019). Expanding the watch list for potential ebola virus antibody escape mutations. PLoS ONE.

[30] Schymkowitz J (2005). The FoldX web server: An online force field. Nucleic Acids Res..

[31] Gonzalez TR, Martin KP, Barnes JE, Patel JS, Ytreberg FM (2020). Assessment of software methods for estimating protein-protein relative binding affinities. PLoS ONE.

[32] Berman HM, Westbrook J, Feng Z (2000). The protein databank. Nucliec Acids Res..

[33] Eastman, P., Swails, J. & Chodera, J. D. OpenMM 7: Rapid development of high performance algorithms for molecular dynamics. *PLOS Comput. Biol.* **13**, (2017). 10.1371/journal.pcbi.1005659 PMC5549999 28746339

[34] Chaudhury S, Lyskov S, Gray JJ (2010). PyRosetta: A script-based interface for implementing molecular modeling algorithms using rosetta. Bioinformatics.

[35] Alford RF (2017). The rosetta all-atom energy function for macromolecular modeling and design. J. Chem. Theory Comput..

[36] Barlow KA (2018). Flex ddG: Rosetta ensemble-based estimation of changes in protein-protein binding affinity upon mutation. J. Phys. Chem. B.

[37] Tyka MD (2011). Alternate states of proteins revealed by detailed energy landscape mapping. J. Mol. Biol..

[38] Lauck F, Smith CA, Friedland GF, Humphris EL, Kortemme T (2010). RosettaBackrub—a web server for flexible backbone protein structure modeling and design. Nucleic Acids Res..

[39] Shapovalov MV, Dunbrack RL (2011). A smoothed backbone-dependent rotamer library for proteins derived from adaptive kernel density estimates and regressions. Structure.

[40] Caswell, T. A. *et al.* matplotlib/matplotlib: REL: v3.5.2. Doi: 10.5281/zenodo.6513224(2022).

[41] Khan A (2021). Higher infectivity of the SARS-CoV-2 new variants is associated with K417N/T, E484K, and N501Y mutants: An insight from structural data. J. Cell. Physiol..

[42] Lu L (2021). The impact of spike N501Y mutation on neutralizing activity and RBD binding of SARS-CoV-2 convalescent serum. EBioMedicine.

[43] Huang H, Zhu Y, Niu Z, Zhou L, Sun Q (2021). SARS-CoV-2 N501Y variants of concern and their potential transmission by mouse. Cell Death Differ..

[44] Colson P (2021). Spreading of a new SARS-CoV-2 N501Y spike variant in a new lineage. Clin. Microbiol. Infect..

[45] Starr, T. N. *et al.* Shifting mutational constraints in the SARS-CoV-2 receptor-binding domain during viral evolution. 2022.02.24.481899 Preprint at Doi: 10.1101/2022.02.24.481899(2022). 10.1126/science.abo7896 PMC9273037 35762884

[46] Cantón R (2021). New variants of SARS-CoV-2. Rev. Esp. Quimioter..

[47] Chakraborty S (2022). E484K and N501Y SARS-CoV 2 spike mutants Increase ACE2 recognition but reduce affinity for neutralizing antibody. Int. Immunopharmacol..

[48] Du P, Gao GF, Wang Q (2022). The mysterious origins of the Omicron variant of SARS-CoV-2. Innovation.

[49] Ma W (2022). Genomic perspectives on the emerging SARS-CoV-2 omicron variant. Genomics Proteomics Bioinform..

[50] Flemming A (2022). Omicron, the great escape artist. Nat. Rev. Immunol..

[51] Cameroni E (2022). Broadly neutralizing antibodies overcome SARS-CoV-2 Omicron antigenic shift. Nature.

[52] Lippow SM, Wittrup KD, Tidor B (2007). Computational design of antibody-affinity improvement beyond in vivo maturation. Nat. Biotechnol..

[53] Beach SS, Hull MA, Ytreberg FM, Patel JS, Miura TA (2022). Molecular modeling predicts novel antibody escape mutations in the respiratory syncytial virus fusion glycoprotein. J. Virol..

[54] Zeng H-L, Dichio V, Rodríguez Horta E, Thorell K, Aurell E (2020). Global analysis of more than 50,000 SARS-CoV-2 genomes reveals epistasis between eight viral genes. Proc. Nat. Acad. Sci..

