# Supplementary material for: The effect of mutations on binding interactions between the SARS-CoV-2 receptor binding domain and neutralizing antibodies B38 and CB6
Source: Sci Rep. 2022 Nov 5;12:18819. doi: 10.1038/s41598-022-23482-5 (PMC9637166; doi:10.1038/s41598-022-23482-5)
Supplement: Supplementary file 3 — Supplementary Information 3. [file 41598_2022_23482_MOESM3_ESM.pdf]

# The effect of mutations on binding interactions between the SARS-CoV-2 receptor binding domain and neutralizing antibodies B38 and CB6f


Jonathan E. Barnes[1], Peik K. Lund-Andersen[1,2], Jagdish Suresh Patel[1,2*], F. Marty Ytreberg[1,3**]

1. Institute for Modeling Collaboration and Innovation, University of Idaho, Moscow, ID 83843
2. Department of Biological Sciences, University of Idaho, Moscow, ID 83843
3. Department of Physics, University of Idaho, Moscow, ID 83843

*thejagdishpatel@gmail.com

***ytreberg@uidaho.edu*


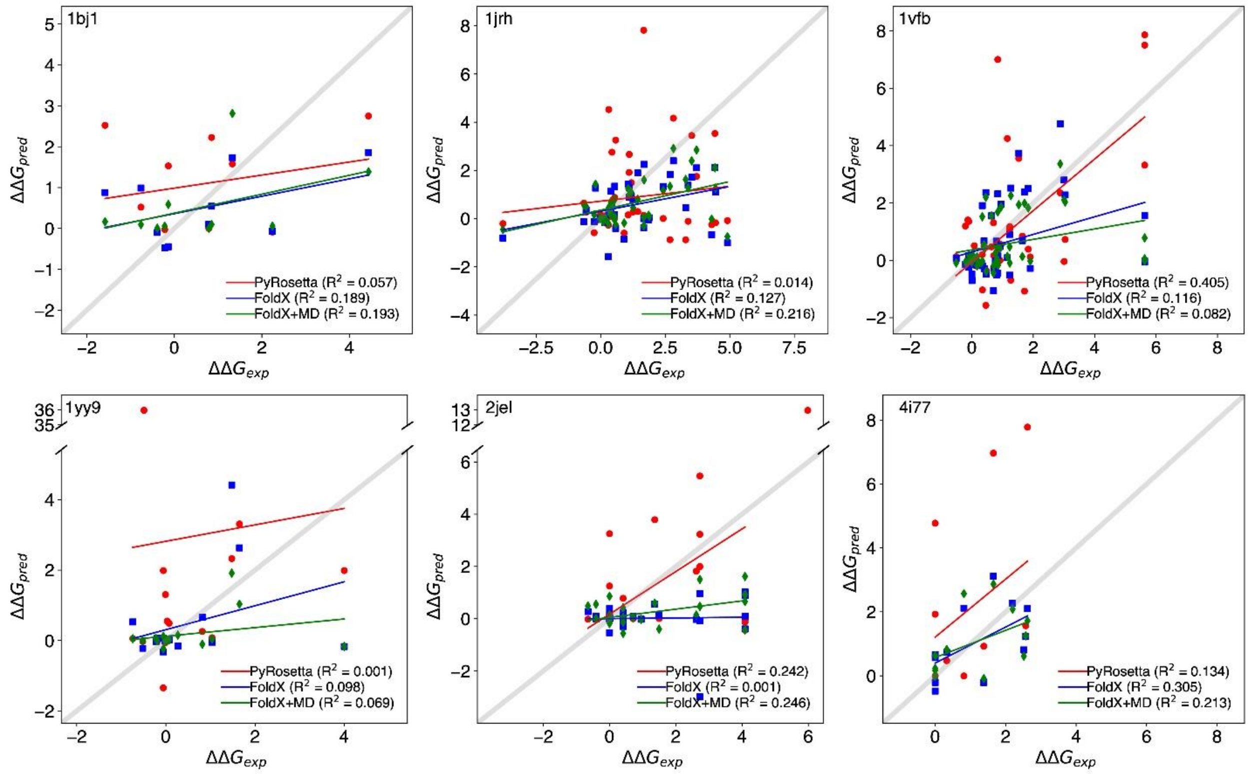


**Supplemental Figure S1** Comparison between FoldX and Rosetta binding affinity predictions for six test systems. The horizontal axis is the experimental data and vertical axis the predicted free energy values. Red indicates Rosetta results, Blue indicates FoldX results, and Green is MD+FoldX results. The grey line is the 1:1 line, if the given methods could predict with perfect accuracy their predictions would fall on this line.

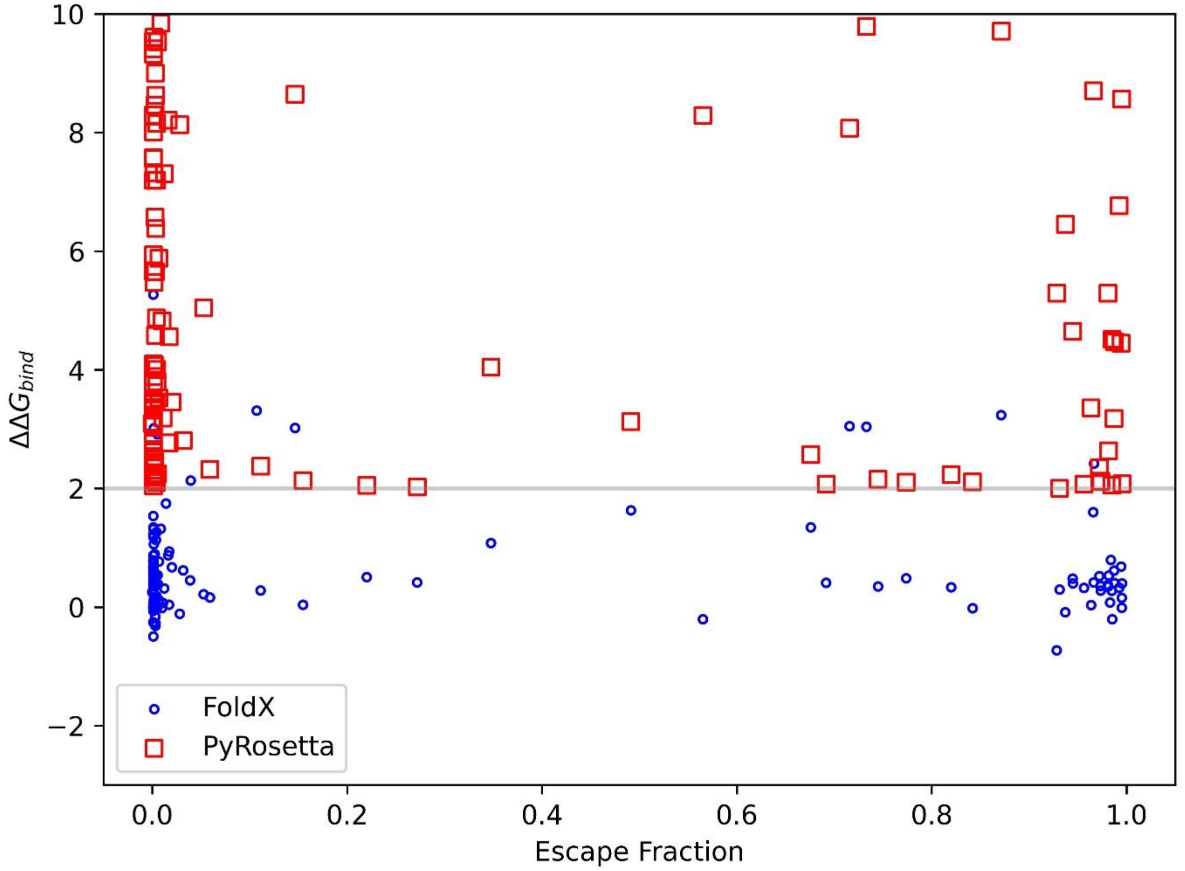


**Supplemental Figure S2** Comparison of FoldX and PyRosetta predictions and escape fraction. The x-axis is escape fraction provided by the raw data from Starr et al[1], this ranges from mutations that lead to no escape (0) to significant escape (1). Binding affinity estimates are shown on the y-axis color coded as blue (FoldX) and red (PyRosetta). The cutoff we chose for escape is indicated with the gray line (2 kcal/mol). There are points above 10 kcal/mol on both sides of the graph from PyRosetta that are extreme outliers and thus excluded from this plot for clarity.

| Site | Number Escape (our data) | Site Total Escape Fraction | Mutations predicted (our data) |
|---|---|---|---|
| E406 | 1 | 0.006927 | Y |
| I418 | 1 | 0.002625 | W |
| D420 | 1 | 7.833 | W |
| S443 | 2 | 0.02766 | W,**Y** |
| V445 | 16 | 0.1308 | **A,E,F,G,H,I,K,L,N,P,Q,R,S,T,W,Y** |
| G447 | 19 | 0.03064 | **A,C,D,E,F,H,I,K,L,M,N,P,Q,R,S,T,V**,W,**Y** |
| L455 | 11 | 4.623 | **A**,D,**F,G,I,P,S,T,V,W,Y** |
| F456 | 18 | 8.844 | **A,C,D,E,G,H,I,K,L,M,N,P,Q,R,S,T,V,W** |
| I472 | 1 | 3.704 | **K** |
| Y473 | 11 | 10.31 | **A,E,F,G,N,P,Q,R,S,T,W** |
| A475 | 18 | 10.29 | **C,D,E,F,G,H,I,K,L,M,N,P,Q,R,T,V,W,Y** |
| G476 | 19 | 3.565 | **A,C,D,E,F,H,I,K,L,M,N,P,Q,R,S,T,V,W,Y** |
| F486 | 5 | 4.421 | **G,K,P,R,S** |
| N487 | 2 | 12.38 | **E**,P |
| Y489 | 3 | 6.851 | E,**I**,T |
| F490 | 3 | 0.1305 | **I,P,V** |
| Q493 | 15 | 1.01 | **A,C,D,F,G,H,K,N,P,R,S,T,V,W,Y** |
| G496 | 5 | 0.1078 | **F,K,R,W,Y** |
| Q498 | 15 | 0.06488 | **A,C,D,E,F,G,H,K,N,P,R,S,T,W,Y** |
| P499 | 1 | 0.1044 | I |
| Y505 | 16 | 0.0653 | A,C,D,E,G,**H**,I,K,L,**M**,**N**,P,**Q**,S,T,V |
| Q506 | 3 | 0.007488 | F,W,Y |

**Supplemental Table S1** Comparison of site level escape predictions compared to site total escape fraction. Column one is our predicted escape sites, column two is the number of mutations predicted to escape at each site, column three is the predicted site level escape fraction from Starr et al[1], column four are the mutations we predict to be escape variants. In column four, bolded values indicate mutations that are in both our dataset and that of Starr et al., non-bold are mutations that we have in ours and were filtered out of Starr's dataset, and underlined are mutations that are only in our dataset, but we also predict to fold.

1. Starr, T. N. *et al.* Prospective mapping of viral mutations that escape antibodies used to treat COVID-19. *Science* **371**, 850–854 (2021).
